# Pulsed Infrared Stimulation of Vertical Semicircular Canals Evokes Cardiovascular Changes in the Rat

**DOI:** 10.3389/fneur.2021.680044

**Published:** 2021-05-28

**Authors:** Darrian Rice, Giorgio P. Martinelli, Weitao Jiang, Gay R. Holstein, Suhrud M. Rajguru

**Affiliations:** ^1^Department of Biomedical Engineering, University of Miami, Miami, FL, United States; ^2^Department of Neurology, Icahn School of Medicine at Mount Sinai, New York, NY, United States; ^3^Department of Neuroscience, Icahn School of Medicine at Mount Sinai, New York, NY, United States; ^4^Department of Otolaryngology, University of Miami, Miami, FL, United States

**Keywords:** optical stimulation, vestibulo-sympathetic reflex, vestibular system, autonomic, heart rate, blood pressure, infrared stimulation

## Abstract

A variety of stimuli activating vestibular end organs, including sinusoidal galvanic vestibular stimulation, whole body rotation and tilt, and head flexion have been shown to evoke significant changes in blood pressure (BP) and heart rate (HR). While a role for the vertical semicircular canals in altering autonomic activity has been hypothesized, studies to-date attribute the evoked BP and HR responses to the otolith organs. The present study determined whether unilateral activation of the posterior (PC) or anterior (AC) semicircular canal is sufficient to elicit changes in BP and/or HR. The study employed frequency-modulated pulsed infrared radiation (IR: 1,863 nm) directed via optical fibers to PC or AC of adult male Long-Evans rats. BP and HR changes were detected using a small-animal single pressure telemetry device implanted in the femoral artery. Eye movements evoked during IR of the vestibular endorgans were used to confirm the stimulation site. We found that sinusoidal IR delivered to either PC or AC elicited a rapid decrease in BP and HR followed by a stimulation frequency-matched modulation. The magnitude of the initial decrements in HR and BP did not correlate with the energy of the suprathreshold stimulus. This response pattern was consistent across multiple trials within an experimental session, replicable, and in most animals showed no evidence of habituation or an additive effect. Frequency modulated electrical current delivered to the PC and IR stimulation of the AC, caused decrements in HR and BP that resembled those evoked by IR of the PC. Frequency domain heart rate variability assessment revealed that, in most subjects, IR stimulation increased the low frequency (LF) component and decreased the high frequency (HF) component, resulting in an increase in the LF/HF ratio. This ratio estimates the relative contributions of sympathetic nervous system (SNS) and parasympathetic nervous system (PNS) activities. An injection of atropine, a muscarinic cholinergic receptor antagonist, diminished the IR evoked changes in HR, while the non-selective beta blocker propranolol eliminated changes in both HR and BP. This study provides direct evidence that activation of a single vertical semicircular canal is sufficient to activate and modulate central pathways that control HR and BP.

Prior studies have shown that vestibular inputs from otolith organs can modulate the autonomic pathway and contribute to the control of blood pressure and heart rate during movement. However, the role of the semicircular canals in this respect has not been completely clear. The present study elucidated the role for the vertical semicircular canals in altering autonomic activity using focused, pulsed infrared stimulation of posterior and anterior canals in the rat. Unilateral activation of either the posterior or anterior semicircular canal was sufficient to evoke profound changes in the heart rate and blood pressure, suggesting that canals also contribute to the vestibulo-autonomic pathway.

## Introduction

The vestibular system is exquisitely sensitive to linear and angular accelerations of the head, informing the nervous system about head position and head movements in space. These two types of signals are detected by different sets of end organs, with the semicircular canals specialized to transduce angular accelerations and the otolith organs detecting linear forces such as gravity, tilt and translation. Vestibular information from both types of end organs is conveyed centrally to participate in well-characterized neural pathways involved in spatial orientation, gaze stabilization, balance, posture and cognition [for reviews, see Holstein (1), Vidal et al. (2)]. In addition, vestibular information is conveyed to regions of the central autonomic nervous system, thereby altering blood pressure (BP), heart rate (HR), and respiration in response to changes in head position and movement [for reviews, see Yates and Miller (3); Yates et al. (4); Yates and Miller (5)]. Since many of these functional pathways specifically target pre-sympathetic brainstem nuclei, they are often referred to as the vestibulo-sympathetic reflex pathways. However, vestibular input to cell groups associated with parasympathetic activity (6) argues for the more general description of the projections as vestibulo-autonomic (7).

The existence and overall functions of the vestibulo-autonomic pathways in humans and animals are now well-established [for reviews, see (Yates et al. (4); Barman and Yates (8)]. For cardiovascular control, the vestibular system provides a rapid and open-loop input to baroreflex pathways mediating changes in peripheral vasoconstriction to compensate for postural adjustments such as standing from a seated or horizontal position (9). Although the baroreflex is an extremely efficient mechanism for maintaining vasomotor homeostasis, it is a closed loop through a long latency negative feedback pathway. In fact, a change in BP following electrical stimulation of baroreceptor afferents requires ~1 s (10). In addition, the baroreflex is entirely reactive, adjusting sympathetic nerve activity to BP perturbations that have already transpired (4).

Most studies of vestibulo-autonomic pathways and functions in humans and experimental animals have focused on the role of the otolith organs in supplying the initial head-related signal. In human subjects, cardiovascular responses evoked by front-back linear acceleration, head pitch, off-vertical-axis rotation and galvanic vestibular stimulation (GVS) (11–23) have typically been attributed to activation of utricular afferents. Similarly, nose-up pitch, head-down tilt, forward linear acceleration, and GVS (24–29) have been utilized in animal models to specifically address the ability of the otolith organs to activate the vestibulo-sympathetic reflex (VSR) and modulate BP. While a contribution from the vertical semicircular canals to vestibulo-autonomic pathways has been suggested previously (30), a specific role for these end organs in central mechanisms of cardiovascular control remains to be identified.

The present study investigated the contributions of the posterior and anterior semicircular canals (PC and AC, respectively) to the vestibulo-autonomic activity using focused, pulsed infrared radiation (IR). This stimulus is advantageous because it offers greater spatial and temporal selectivity than electrical activation (31–34). In the vestibular system, pulsed IR has been shown to rapidly alter the rate of transmitter release from inner ear hair cells and modulate the discharge rate of primary afferent fibers (34–36). In addition, our group recently reported that pulsed IR can be used to selectively activate the PC in anesthetized rats (37). In the present study, we measured eye movements, BP, HR, and heart rate variability (HRV) during unilateral pulsed IR stimulation of the PC or AC. The findings provide new evidence directly linking activation of the vertical semicircular canals to changes in HR and BP.

## Experimental Methods

### Animal Preparation and Surgical Approach

**Figure 1:**
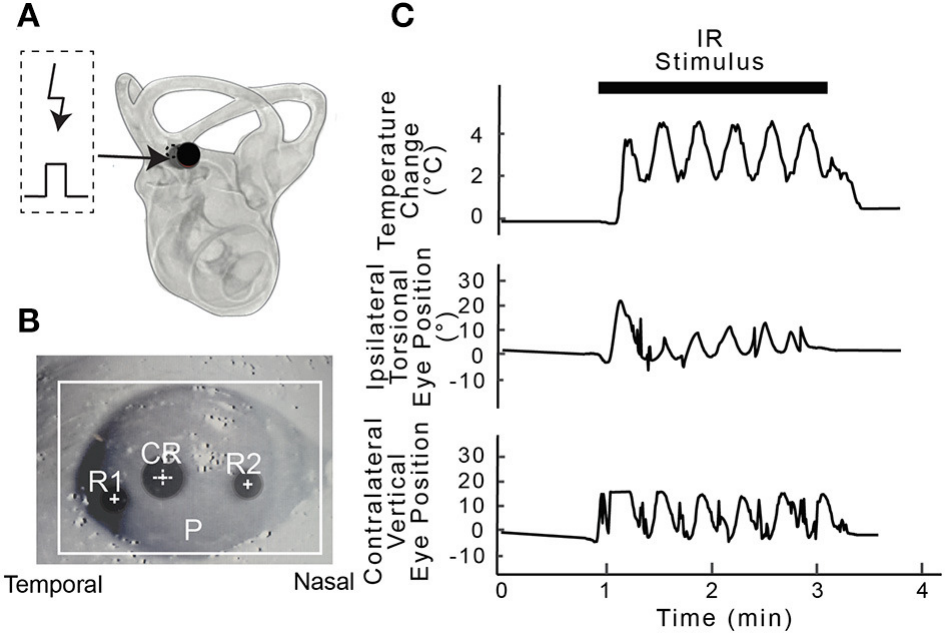
Experimental setup. **(A)** Shows an example post-mortem microtomography image of the right inner ear of a rat with a 400 μm optical fiber (solid circle) used for IR stimulation targeting the PC ampulla. A micro thermistor (dotted circle) was inserted beside optical fiber and used to measure the change in temperature along the beam path during posterior canal stimulation. **(B)** Eye recording setup highlighting pupil (P), cornea reflection (CR) and rotational marks (R1 and R2) was used to calculate the magnitude of the movement during IR stimulation. **(C)** Simultaneous physiological recordings were carried out during each 2-min IR stimulation period. The panels show IR stimulation of the vestibular end organs at 0.5 Hz, the change in temperature along the beam path (°C), and frequency-matched ipsilateral torsional and contralateral vertical eye position change (°) from IR stimulation of the PC in one rat.

All experiments were carried out in accordance with the National Institutes of Health Guide for the Care and Use of Laboratory Animals and the Institutional Animal Care and Use Committee (IACUC) of the University of Miami approved all procedures. In total, 30 adult male Long Evans rats (Charles River Laboratories) weighing 300–500 g were used in this study. Infrared stimulation of the PC was attempted in 23 animals including the pharmacological studies, PC electrical stimulation results were obtained from an additional three animals, and IR evoked responses to AC IR stimulation were obtained from four rats. The experimental approach and examples of the physiological data obtained during the experiments are illustrated in Figure 1.

### Surgical Approach

For stimulation experiments, animals were initially anesthetized with intraperitoneal injections of ketamine (44 mg/kg) and xylazine (5 mg/kg). Anesthesia was maintained with supplemental doses of these agents, based on assessments of the animal's pinch reflex tested every 15 min throughout the experimental session. Vital signs were continuously monitored, and the core body temperature was maintained at 37°C by resting the animal on a heating pad (Deltaphase® Isothermal pads; Braintree Scientific) throughout the procedure. Body temperature was monitored using the telemetric system described below. Prior to stimulation, all rats were anesthetized (as above), fitted with head posts and secured on a custom-designed stereotaxic system (Kopf Instruments). To accomplish this, a 2 cm skin incision was made over the skull and the bony surface was exposed and cleaned. Two surgical screws were anchored into symmetrically-placed holes drilled in the parietal bones. A stainless-steel head post was fitted between the screws and secured in place by dental acrylic cement (Jet Repair). To access the vestibular end organs, a C-shaped incision was made behind the right pinna and blunt dissection was performed to remove the surrounding muscles and expose the bulla. The bulla was opened using a motorized surgical drill in order to visualize the round window and direct the laser stimulation toward the PC ampulla. To target the AC, the middle ear ossicles were carefully removed with forceps and the AC ampulla was identified using local anatomical landmarks (38).

The animals were placed on the stereotaxic frame and secured using the head post to maintain immobility during the stimulation experiment. Note that the bony labyrinth covering the crista ampullaris was not opened since IR directed at the canal crista through the thin bone was sufficient to evoke physiological responses. Confirmation that the end organs were accurately targeted by the IR stimulation was based on evoked eye movements and by post-mortem microtomography (37). For the microtomography, following the terminal experiment the inner ears were fixed in 4% paraformaldehyde in 0.1 M phosphate buffer with the optical fiber fixed in place using dental acrylic. The tomographic images taken of the samples (Skyscan 1176 Micro Photonic Inc.) were imported in Osirix Lite (Pixmeo) for 3-D reconstruction, low-pass filtered and the fiber orientation relative to the AC or PC crista was determined from the three-dimensional reconstructions (Figure 1A).

### Infrared Stimulation

Pulsed IR stimulation (1,863 nm wavelength) was achieved with a Capella diode laser (Lockheed Martin Aculight Corp.) and frequency modulated by external function generators (Tektronix, mode CFG250, AFG320) where *f* = *f*0 ((1 − *a*) + *a* sin (2π*fst*)); the baseline frequency was *f*_0_ = 250 Hz, the modulation frequency was *f*_s_ = 0.05 Hz, and the dynamic range was *a* was fixed at 0.9. The laser was coupled to a 200 or 400 μm optical fiber (P200-5-VIS-NIR and P400-5-VIS-NIR, Ocean Optics) to target the vestibular neuroepithelium. IR with a wavelength of 1863 nm, frequency of 250 pulses per second (pps), and pulse duration of 200 μs, was delivered sinusoidally at 0.05 Hz to the right posterior or anterior ampullae for 2 min. The penetration depth used in the present study was ~0.8 mm in water (39). Only the radiant energy was varied between 20 and 149 μJ/pulse for the 200 μm fiber and between 94 and 512 μJ/pulse for the 400 μm fiber to determine dose-dependent stimulation-evoked changes in BP and HR. The output radiant energies reported in the results were characterized from end-polished optical fibers in air (40). These energy values likely vary from those experienced *in vivo* at the target tissue depending upon the distance between the fiber tip and the target tissue, thickness of the bone, and/or the beam path. In one rat, local temperature at the crista during IR stimulation was recorded using a micro-thermocouple (Omega) placed in the path of the IR optical beam. The temperature was recorded at a sampling rate of 60 Hz for three 2-min stimulation periods (Figure 1C).

### Electrical Stimulation

To further confirm the involvement of the vertical semicircular canals in the evoked BP and HR changes, electrical stimulation of the PC was performed in 3 rats. The surgical approach to the bulla and round window were as described above for the IR stimulation. In each rat, a standard bipolar needle electrode was placed at the site of the PC ampulla and a second electrode was placed at the upper rim of the round window. Monophasic pulses of 500 μs pulse duration, 250 pps, and 700 μA per pulse were generated by an isolated stimulator (ISO-STIM 01M, NPI) and sinusoidally modulated at 0.05 Hz, a frequency that was used in the IR experiments. The total duration for electrical stimulation was 2 min, consistent with the IR stimulation.

### Eye Movements

Activation of the targeted end organ—PC or AC—during either infrared or electrical stimulation was confirmed using eye movements (37, 41) that were observed and recorded in some animals using a video-oculography system (ETL-200; ISCAN Inc., Figure 1B and lower traces in Figure 1C). The system located and tracked the center of the pupil and corneal reflections of both eyes. The linear positions of the pupil and corneal reflection were converted to angular rotation in the horizontal and vertical directions using previously detailed methods (37). Sinusoidally-modulated IR stimulation of the PC evoked frequency-matched torsion in the ipsilateral eye and a vertical downward movement in the contralateral eye whereas that of the AC evoked primarily upward movements ipsilaterally and upward extorsion contralaterally. In majority of the rats, eye movements continued to modulate for the duration of the experiment.

### Sensor Implantation

During the stimulation experiments, HR, BP, and body temperature were measured with a small animal single pressure device (DSI Pressure Sensing Technologies, model HD-S10) implanted in the femoral artery prior to stimulation. Implantation surgeries were conducted at least 24 h before the IR experiments using aseptic technique. Animals were anesthetized with isoflurane (2–3%) using a portable anesthesia workstation (Eagle Eye Anesthesia). The telemetry device was implanted according to procedures reported previously (42). Briefly, the insertion area above the left groin was shaved and cleaned with a surgical antiseptic (Providone-iodine), and then a small incision was made to expose the femoral artery. An arteriotomy was performed and the transducer catheter was inserted into the vessel. The artery was tied off to secure the catheter and the body of the sensor was secured in a subcutaneous pouch. The incision was closed with a suture and surgical staples. Post-surgical pain was monitored and managed with buprenorphine (0.3 mg/ml). Animals recovered for a minimum of 24 h before they were used in stimulation experiments.

### BP and HR Recordings

Physiological data were collected continuously using the implanted single pressure telemetric sensor and Ponemah software (DSI). Using this system, arterial pressures were recorded continuously and analyzed every 5 s to calculate parameters including: systolic BP, diastolic BP, mean BP, HR, and body temperature. BP and HR recordings were initiated prior to the stimulation in order to establish the baseline activity for each animal, and continued throughout the experiment. The start and end times for each 2-min IR stimulus were marked as trigger events in the recordings to facilitate the subsequent data analysis. Data exported from Ponemah as CSV files were analyzed with custom scripts written in MATLAB (Mathworks).

### Heart Rate Variability Analysis

The inter-beat intervals of arterial BP signals were analyzed using the Ponemah (DSI) software to calculate heart rate variability (HRV). Using Fast Fourier transforms to perform the spectral analysis, HRV was divided into components that operate in different frequency ranges (43, 44). The HRV frequency bands used in rat are: Very Low Frequency (VLF) [0.05–0.25 Hz], Low Frequency (LF) [0.25–1 Hz], and High Frequency (HF) [1–3 Hz]. Our HRV data are presented as the ratio between normalized LF and HF, which excludes the VLF data including the IR stimulation frequency 0.05 Hz. As noted above, HRV measures were initially obtained for the overall baseline BP recorded prior to any IR stimulation. Baseline HRV measures were then confirmed from the 2 min BP recordings obtained immediately preceding each IR stimulation trial. The changes recorded during each 2 min stimulation trial were compared to the HRV during the baseline (no stimulation) condition. The data compiled in Ponemah (DSI) were exported to CSV files and further analyzed and plotted in MATLAB (Mathworks, MA). The ratio of LF to HF power was also used to estimate the relative ratio of sympathetic to parasympathetic nervous system activity (43).

### Pharmacological Blockers of Autonomic Function

In six experiments, we evaluated the contributions of sympathetic and parasympathetic activity to the vestibulo-autonomic responses evoked by IR. The muscarinic cholinergic receptor antagonist atropine (1 mg/kg) or the non-selective beta-adrenergic receptor blocker propranolol (1 mg/kg) were administered systemically via the tail vein in anesthetized rats. Baseline and IR-evoked changes in BP and HR were recorded prior to and following administration of the drug. Pulsed IR (0.05 Hz) stimulation of the PC was performed as above. IR evoked responses were compared (a) prior to drug injection, (b) after the BP and HR baselines stabilized following drug administration, and (c) 40–50 min following drug administration to determine whether the BP and HR responses recovered.

## Results

### IR Stimulation of the PC Modulates HR and BP

Unilateral stimulation of the PC with pulsed IR of wavelength 1,863 nm induced changes in eye position that matched the stimulus frequency (0.05 Hz, in 17 rats). The torsional, vertical movement of the ipsilateral (right) eye and downward movement of the contralateral (left) eye that are characteristic of PC stimulation (37) were confirmed visually in all rats. Figure 1C shows an example of the evoked sinusoidal eye movements from one of the rats that also provided the HR and BP data. The eye movements were used to confirm positioning of the optical fiber to target the PC sensors.

**Figure 2:**
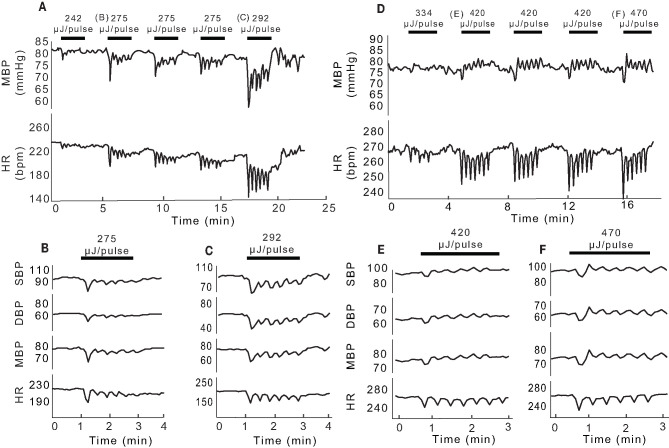
Changes in BP and HR evoked by unilateral pulsed IR directed at PC. **(A)** Shows example mean BP (mmHg) and HR (bpm) responses recorded in one rat at different IR radiant exposures. Black bars indicate the 2-min IR stimulus intervals with the radiant energy at each interval noted above (242 to 292 μJ/pulse). The initial observable response (threshold) occurred at 242 μJ/pulse. **(B,C)** Show single events at 275 and 292 μJ/pulse, respectively and the corresponding changes in systolic (SBP), diastolic (DBP), and mean (MBP) pressures as well as changes in HR. **(D)** Shows responses recorded in another rat at different radiant exposures (292 to 470 μJ/pulse) with the observed threshold at 420 μJ/pulse. **(E,F)** Show single events at 420 and 470 μJ/pulse, respectively, and the corresponding changes in SBP, DBP, MBP, and HR.

In the experiments highlighted in Figure 2, IR was delivered via a 400 μm optical fiber and the radiant energy of the stimulation was varied. At each energy level, decreases in HR and mean BP were observed at the onset of the sinusoidal stimulation (Figure 2A). As the IR stimulation proceeded over the 2-min periods (black bars, Figure 2A), the BP and HR modulated with the stimulus, and returned to baseline when the laser power was switched off. Multiple 2-min activation periods using the same stimulus conditions evoked similar responses during an experiment. For the example shown in Figure 2A, the threshold radiant energy required to elicit a change in BP or HR was 242 μJ/pulse. Subsequent stimuli at higher radiant energies, 275 and 292 μJ/pulse in this rat, resulted in characteristically similar, but larger BP and HR responses. Both physiological responses continued to modulate sinusoidally, matching the 0.05 Hz IR stimulation frequency. In this experiment, the initial decrease in BP ranged from 3.5 to 19 mmHg and initial decrease in HR ranged from 7.5 to 70 bpm, depending on the radiant energy of the pulses. HR and mean, systolic and diastolic BP changes in response to pulses of two different energy levels are shown on an expanded time scale in Figures 2B,C.

Figures 2D–F illustrate data from a second rat, in which the threshold IR radiant energy required to elicit a change in BP and HR was notably higher at 420 μJ/pulse (Figure 2D). In this animal, HR decreased at the onset of IR and remained below the baseline for the duration of IR stimuli. However, BP decreased transiently at the onset of IR stimulation and continued to modulate at low amplitude near the baseline. At the cessation of IR, both BP and HR returned to baseline and remained stable until the next stimulus. The initial reduction in BP ranged from 4.06 to 7.24 mmHg and that of HR ranged from 23.65 to 34.13 bpm across the five stimuli shown in Figure 2. Figures 2E,F show the changes in HR and mean, systolic and diastolic BP for 2-min stimulation periods at the threshold of 420 μJ/pulse and at a higher radiant energy of 470 μJ/pulse, respectively. Consecutive stimulation cycles continued to evoke characteristically similar responses.

**Figure 3:**
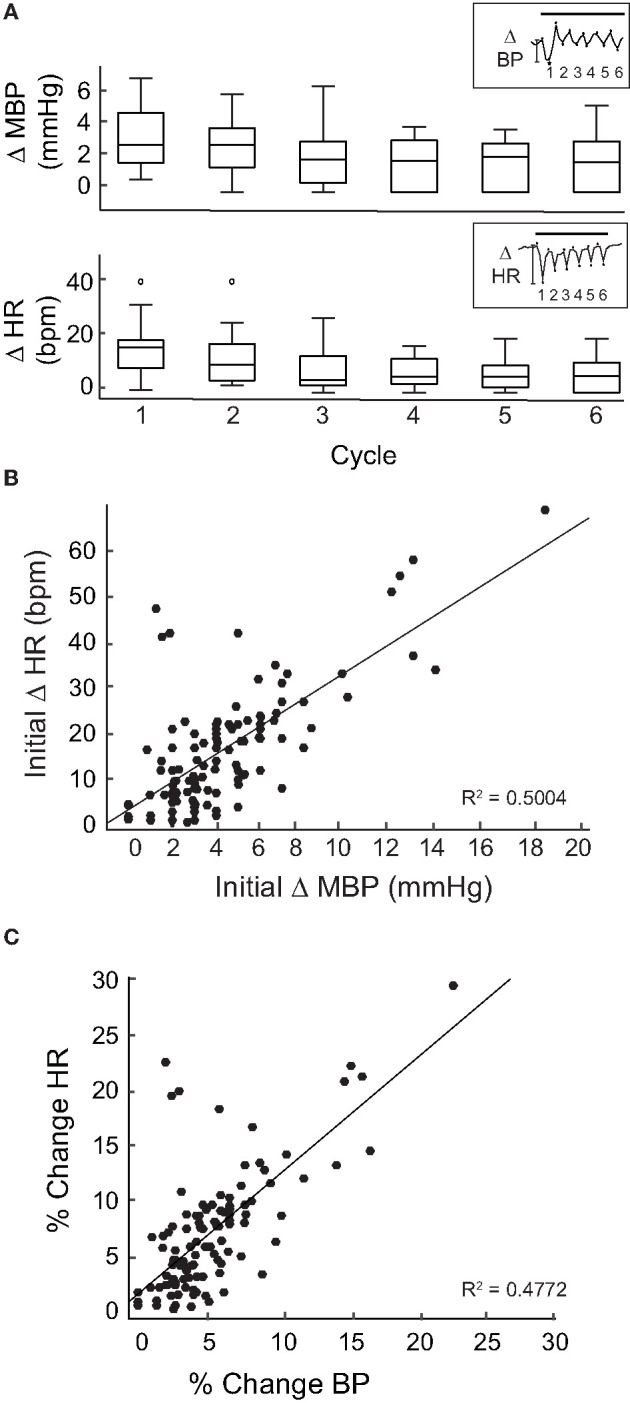
Magnitude of change per cycle. **(A)** Shows a box plot of the changes in MBP (top) and HR (bottom) for each cycle (20 s) of the 2-min stimulation period at 470 μJ/pulse in 6 rats. Inset shows one example of a MBP (top) and HR (bottom) measurement during a stimulation event at 470 μJ/pulse divided into 6 cycles. The magnitude of change was measured peak-to-peak (*) for each cycle. Change in MPB and HR between cycles was not significant [repeated measures ANOVA: HR (*p* = 0.3644) and BP: (*p* = 0.7952)]. **(B)** The initial change in MPB (mmHg) from 113 events in 17 rats correlated moderately to the corresponding initial changes in HR (bpm) (*R*^2^ = 0.5004, *p* < 0.0005). **(C)** Percent change was taken for both MBP (initial change MBP/MBP baseline) and HR (initial change HR/baseline HR) at 113 stimulation events across 17 rats. The percent changes in HR and MBP correlated moderately (*R*^2^ = 0.4772, *p* < 0.0005).

Overall, similar HR and BP responses were obtained from 17 rats in 113 IR stimulation trials of the PC. Across animals, the general profiles of the transient responses during IR were similar and IR stimulation evoked responses in each rat. However, the amplitude of the initial and subsequent decreases in BP and HR varied across animals and across stimulation trials. To determine whether the evoked changes decreased over the course of the stimulation period, we analyzed the peak-to-peak BP and HR responses over six intervals or cycles within the 2-min IR stimuli. The initial trough was taken as the first lowest value of HR or MBP when compared with an averaged baseline. Subsequent peaks/troughs were defined by the next highest/lowest values of MBP and HR when compared with the previous peak or trough. Responses from six rats in which IR stimulation was carried out for a cumulative total of 12 times at the same radiant energy (470 μJ/pulse) were pooled. The change in HR during the first stimulus cycle ranged from 1 to 43 bpm with a median of 17.47 bpm, while that for Cycle 6 ranged from 0 to 21 bpm with a median of 6.39 bpm (Figure 3A). The change in BP during the first cycle ranged from 1 to 7.24 mmHg with a median of 3 mmHg, and from 0 to 5.53 mmHg, median of 1.94 mmHg, for the sixth cycle. These cycle-to-cycle changes in BP and HR were not statistically significant (repeated measures ANOVA: HR, *p* = 0.3644 and BP, *p* = 0.7952).

Next, we focused on the initial declines in mean BP and HR at the onset of IR stimulation. When these magnitudes were calculated for each of the 113 IR stimuli delivered to the 17 rats, a moderate correlation was observed between magnitudes of the initial changes in mean BP and HR (*R*^2^ = 0.5004, *p* < 0.0005, Figure 3B). To further investigate this, the percent changes in BP and HR were calculated by dividing the magnitude of change (initial decrease) in BP or HR for each IR stimulation period by the pre-stimulation baseline BP or HR for each rat. We found that these percent changes in BP and HR also correlated moderately (*R*^2^ = 0.4772, *p* < 0.0005, Figure 3C) Supplementary Figure 1 shows the relationship between percent change in BP and percent change in HR for each of the rats that received PC stimulation. These data show that, despite the variability in responses between individual animals, the overall correlation between changes in HR and changes in BP during IR stimulation is moderate.

**Figure 4:**
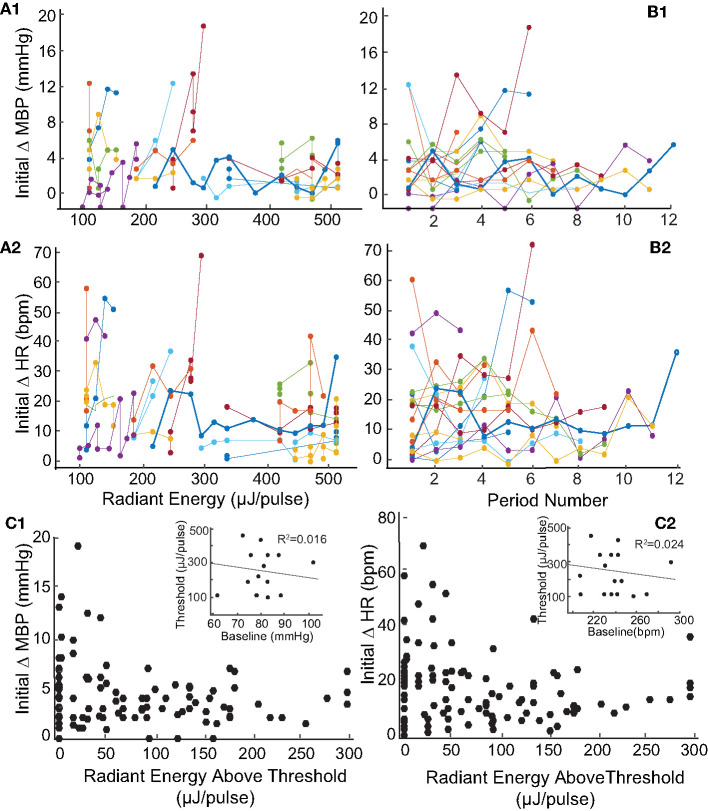
Relationship between magnitude of change in blood pressure and heart rate and radiant energy or IR stimulation trials. **(A)** There is a no correlation between stimulation radiant energies (μJ/pulse) and the initial changes in **(A1)** MBP (mmHg) (*r* = −0.331) and **(A2)** HR (bpm) (*r* = −0.269). The results shown are from 113 IR stimulation events across 17 rats. **(B)** Shows the relationship between consecutive IR stimulation periods and the magnitude of change in **(B1)** MBP (mmHg) (*r* = −0.0647) and **(B2)** HR (bpm) (*r* = −0.0725) in 17 rats. The responses over multiple IR stimuli for individual rats are represented using different colors. **(C1,C2)** The initial changes in both parameters with stimuli radiant energy normalized to that at thresholds are shown. The insets show a weak correlation of threshold radiant energy required to evoke a response to baseline MBP or HR.

Like the two examples shown in Figure 2, the levels of IR radiant energy that were required to elicit BP and HR changes varied across the 17 rats. To examine this further, we sought to determine whether the radiant energy per IR pulse (strength of the stimulus), the compilation of stimuli (multiple trials in the same rat), or baseline physiology of the rat affected BP or HR. Across the 113 IR stimuli applied to the 17 rats with PC stimulation, radiant energy varied between 72 and 512 μJ/pulse. Figure 4 shows the magnitude of changes in BP (Figure 4A1) and HR (Figure 4A2) evoked by IR across different radiant energies per pulse in each of the 17 rats. The BP decreased between 1 and 19 mmHg with a median of 4 mmHg. However, these decrements in BP did not correlate with increasing radiant energy in a dose-dependent manner (*r* = −0.331). Similarly, HR diminished between 1 and 68 bpm with a median of 15 bpm, and also did not correlate with increasing radiant energy (*r* = −0.269). Each IR stimulation was 2 mi in duration and in most of the rats, the stimuli were repeated several times over 60–90 min. On average, 7 IR consecutive stimulation trials were applied per rat. To investigate potential adaptation or additive effects with repeated stimulation, the initial changes in BP and HR evoked at IR onset were compared across stimulation trials (Figures 4B1,B2). Neither the BP decrease (*r* = −0.0647) nor the fall in HR (*r* = −0.25) correlated with the number of consecutive IR stimuli or with the radiant energy required to evoke the response above threshold. The threshold radiant energy required to elicit a change in BP or HR had a broad range, from 94 to 445 μJ/pulse (224 ± 118 μJ/pulse, mean ± SD, Figures 4C1,C2, insets).

**Figure 5:**
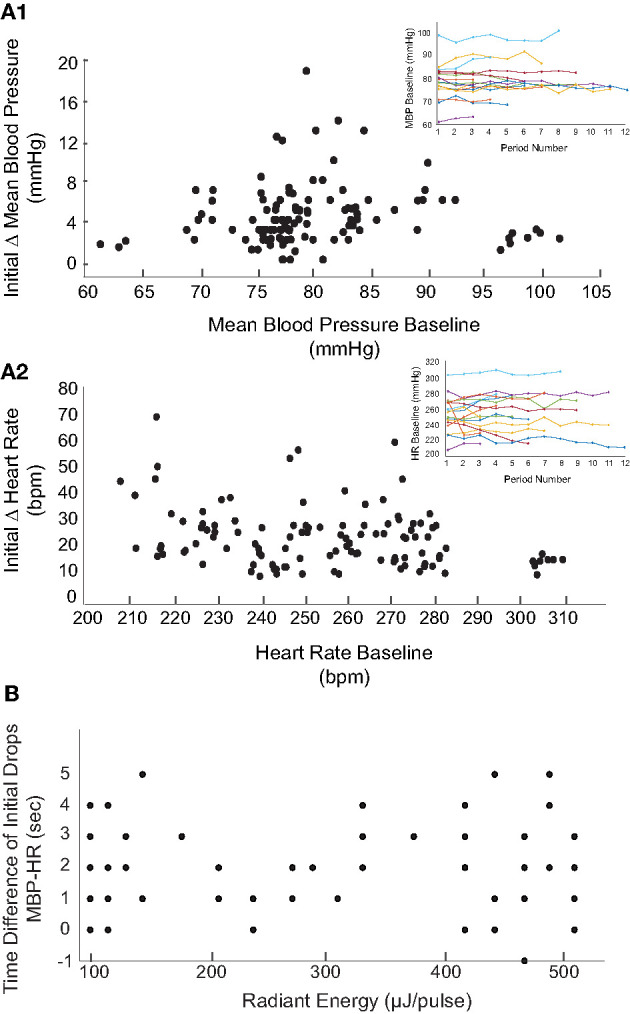
Relationship between baseline and magnitude of changes in BP and HR. The initial decreases in MBP **(A1)** and HR **(A2)** in response to the 113 IR stimulation events (17 rats) are shown with respect to the averaged baseline MBP and HR prior to each stimulation. Overall, MBP and HR remained steady throughout the experiment (insets show the MBP and HR baselines over multiple IR periods for the 17 rats). **(B)** The relationship between latency of responses to IR at varying radiant energies (μJ/pulse) is shown. Latency of responses were calculated by taking the difference in time (seconds) of the initial decrease in MBP from initial decrease in HR.

The baseline BP (82.3 ± 8.6) and HR (244.9 ± 22.7) prior to stimulation varied significantly between the animals. The baseline physiological responses can be affected by the duration of surgeries, effects of anesthesia (including multiple dosages), and inter-subject physiological variations. To determine whether the baseline physiology at the time of IR stimulation affected the evoked changes, the IR-evoked changes in mean BP and HR were compared to pre-stimulation baseline measurements. These results are summarized in Figure 5. The baseline measurements were obtained from averaged BP and HR recorded over a 2-min duration prior to each IR stimulation trial in each rat. The baseline MBP and HR in the 17 rats did not vary significantly over the duration of experiments (Figure 5, insets). Across the 113 trials in the 17 rats, the averaged baseline BP ranged from 61 to 102 mmHg (82.3 ± 8.6) while the BP changes during the first cycle of IR stimulation ranged from 1 to 19 mmHg. The largest changes in BP were observed in rats in which the baseline BP was within a physiological range of 72 to 90 mmHg. The baseline HR varied significantly, ranging from 208 to 311 bpm, while the HR decreases during the first cycle of IR stimulation ranged from 7 to 71 bpm.

**Figure 6:**
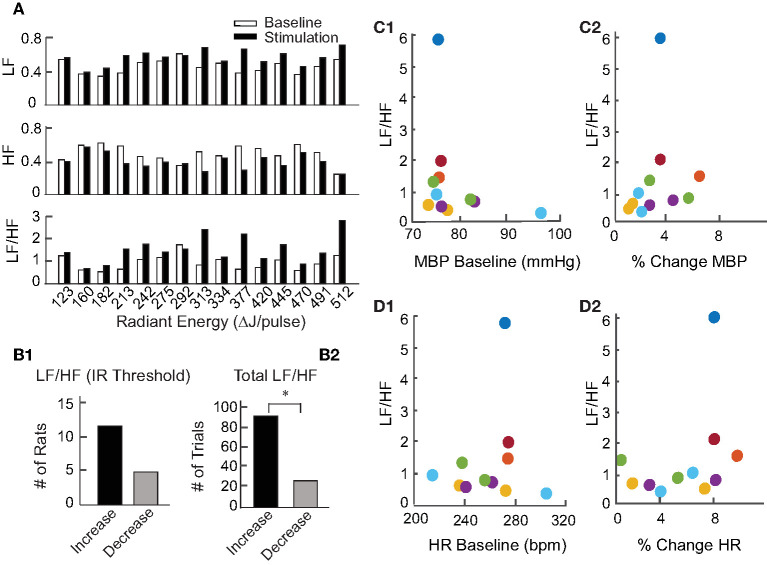
Heart rate variability during pulsed IR targeting PC. **(A)** Shows an example of HRV changes in the LF, HF, and LF/HF components evoked by IR in one rat. Baseline responses, shown by white bars, were recorded for 2 min prior to initiation of IR. Responses to stimulation, shown by black bars, were recorded for 2 min during stimulation of the PC by IR. **(B1)** Shows the number of rats with an increase or decrease in LF/HF at each of the 17 rats' stimulation threshold. **(B2)** Shows the number of stimulation periods that resulted in either an increase or decrease in LF/HF over all periods (113) in 17 rats, (sign test, *p* < 0.001). **(C1,D1)** Show the relationship between HRV (LF/HR ratio) and baseline levels of MBP or HR for responses to IR stimulation of the PC at 470 μJ/pulse in 6 rats which are color coded. **(C2,D2)** Similarly show the relationship between HRV (LF/HF ratio) and percent change in MBP (initial change MBP/MBP baseline, or percent change in HR (initial change HR/baseline HR) for responses to IR stimulation of the PC at 470 μJ/pulse in the 6 rats.

To investigate the physiological changes underlying the reductions in BP and HR upon IR stimulation, heart rate variability (HRV) was analyzed for all animals. In the frequency domain, spectral analysis of the inter-beat interval was used to divide the variability into VLF (0.05–0.25 Hz), LF (0.25–1.0 Hz), and HF (1.0–3.0 Hz) components. The LF and HF components were normalized to remove VLF components for each stimulation event across the 17 rats, thereby eliminating the potential power contributions at the 0.05 Hz IR stimulation frequency. The LF/HF ratios were then calculated to assess HRV over the 2 min prior to stimulation (baseline) and for 2 min during stimulation at each radiant energy level. Figure 6A shows one example comparing the LF, HF, and LF/HF ratios at baseline and during IR-evoked responses. In this experiment, IR was delivered in 15 trials with increasing steps of radiant energy ranging from 123 to 512 μJ/pulse. In 11 of 17 animals, including this example (Figure 6B1), the LF components increased, HF components decreased, and the resulting LF/HF ratios increased during the IR-evoked responses. Of 113 IR trials, 90 resulted in an increase in the LF/HF ratio whereas only 23 resulted in a decrease (Figure 6B2). Additionally, we investigated the relationships between the frequency components (LF and HF) and pre-stimulation BP and HR baselines (Figure 2) as well as the relationships between the frequency components and baselines (Figures 6C1,D1) or percent changes (Figures 6C2,D2) in BP and HR in animals that were stimulated at a single radiant energy: 470 μJ/pulse. Neither component was significantly correlated with resting hemodynamic parameters or the IR induced total change in these parameters.

We also investigated the latency of PC- stimulation-induced cardiovascular responses across all 113 stimulation events (radiant energy varying from 105 to 512 μJ/pulse). The latency was calculated as the time required for the BP or HR to decrease to the lowest measured value following IR onset. The BP and HR latencies in 12 trials at a single radiant energy (470 μJ/pulse) demonstrate that the decrease in HR at this relatively high IR radiant energy occurred 1.42 ± 1.62 s prior to the reduction in MBP. At this radiant energy, the latency to the first drop in MBP was 16 ± 4.4 s whereas for HR was 14.4 ± 5.03. These findings are summarized in Figure 5B and reported as the time difference between the initial decrease in MBP and the initial decrease in HR. Time differences greater than zero indicate that the decrease in HR occurred prior to the decrement in MBP. Across the 117 trials, the reduction in HR occurred on average 2.05 ± 1.51 s prior to the decrease in BP. There was no correlation between these latencies and the radiant energy per pulse delivered during the IR stimulation.

### HR and BP Responses to Electrical Stimulation of the PC

**Figure 7:**
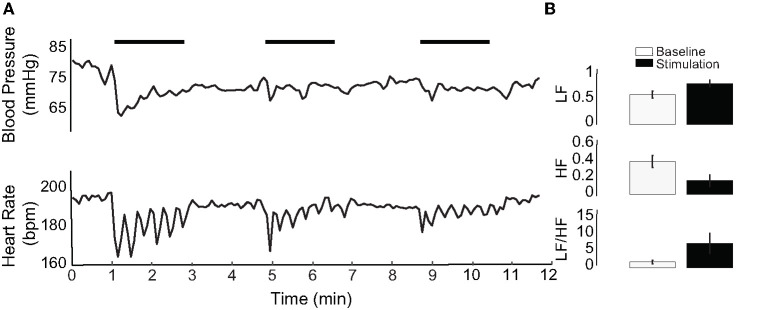
Electrical stimulation of the PC. **(A)** An example of electrical stimulation in one of three stimulated rats. IR stimulation periods are indicated by horizontal black bars. **(B)** Shows HRV of the electrically stimulated rat in **(A)**, averaged over three stimulation periods. LF increases, HF decreases, and the LF/HF ratio increases with electrical stimulation of the PC.

Our previous research has shown that IR of the vestibular neuroepithelium evokes diverse post-synaptic afferent spike trains with inhibition, excitation, and/or phase-locked responses (34, 36). The photothermally driven mechanism of IR stimulation in cells also leads to local temperature increases at higher radiant energies (36, 45) (Figure 1A). The IR stimulation used in the present study was also unilateral, and as such not a physiological stimulus condition. To assess the reliability of the evoked physiological responses, the PC was stimulated with frequency-modulated electrical currents in three naïve rats (no prior IR stimulation). Monophasic pulses of 500 μs phase duration and 700 μA per pulse were sinusoidally modulated at 0.05 Hz for three consecutive 2-min intervals matching the IR stimulation paradigm. The electrically-evoked BP and HR responses are shown in Figure 7A for one animal during three successive trials. Across these trials, the initial decrease in BP varied between 6.41 and 16.68 mmHg, and the reduction in HR varied between 7.3 and 33.63 bpm. Responses to 9 electrical stimulation trials in the three rats (three trials per rat) were obtained. Overall, the BP decrements ranged between 1.28 and 18.13 mmHg and those of HR varied between 3.84 and 72.78 bpm. In each of the three animals (example in Figure 7B), the HRV analysis was characteristically similar to that resulting from IR stimulation: the LF components increased and HF components decreased. All nine electrical stimulation trials resulted in an increase in the LF/HF ratio (Figure 7B), matching the majority of responses to IR stimulation of the PC.

### IR Stimulation of the AC Modulates HR and BP

**Figure 8:**
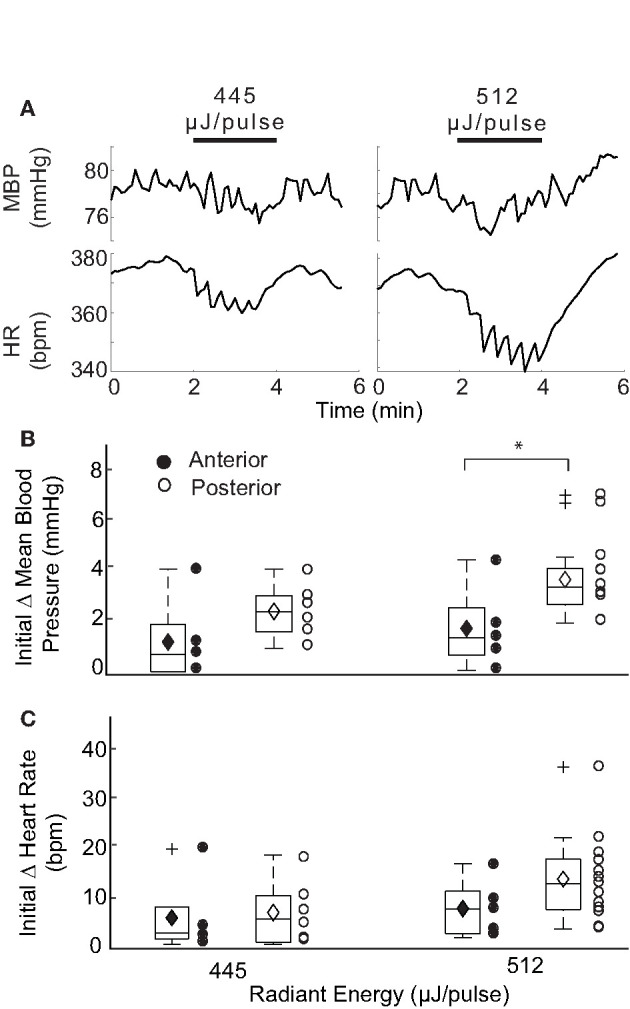
Responses evoked with IR stimulation of the AC vs PC. **(A)** Shows two example responses recorded in one rat at different IR stimulation of the AC. Changes in mean BP and HR were recorded for cycles stimulated at 445 and 512 μJ/pulse. The initial decrease in BP at the higher radiant energy of 512 μJ/pulse was determined to be 0.81 mmHg. Initial decreases in HR were observed to be 3.49 and 8.17 bpm at respective radiant exposures. Box plots show comparison of the initial changes in MBP **(B)** and HR **(C)** between IR stimulation of AC (closed circle) and PC (open circle) at two different energy levels: 445 and 512 μJ/pulse. A significant difference in the responses evoked by IR stimulation of the two canals was observed in MBP and only at 512 μJ/pulse [P(T≤t) two tail = 0.047].

The IR parameters described above were used to stimulate the AC in 4 naïve rats (13 trials) while measuring changes in BP and HR. Stimulation of the AC was confirmed in each rat by the characteristic eye movements evoked in response to IR (37). Figure 8A shows an example of the reductions in HR and BP evoked by IR stimulation of the AC in two trials. Despite significant fluctuations in the BP response, an initial decrease in HR and sinusoidal modulation with pulsed IR were observed. We were unable to determine an initial decrement in BP using a radiant energy of 445 μJ/pulse, although an initial decrease in HR of 3.49 bpm occurred. Using higher stimulus energy (512 μJ/pulse), an initial reduction in BP from baseline levels of 0.81 mmHg was observed, together with a 8.17 bpm decline in HR. Both BP and HR returned to baseline post-IR, and similar responses were observed in all 4 rats.

A comparison of the responses evoked by the two vertical semicircular canals is summarized in Figures 8B,C for mean BP and HR at two different radiant energies. Overall, IR stimulation of the AC evoked MBP changes ranging from 0.65 to 3.94 (median 1.11) at 445 μJ/pulse and 0.81 to 4.33 (median 1.56) at 512 μJ/pulse. AC evoked decrements in HR ranged from 1.22 to 19.82 (median 3.49) at 445 μJ/pulse and 2.82 to 16.54 (median 8.17) at 512 μJ/pulse. These responses modulated with the IR stimulation and were characteristically similar to those observed with PC stimulation. Significant differences between the AC- and PC-evoked responses were found only for the initial reduction in MBP at 512 μJ/pulse (*p* = 0.047, two-tail *t*-test). The AC-evoked responses were also analyzed to examine the relationships between magnitude of change and pre-stimulation baselines as well as the radiant energy and consecutive stimulation periods Supplementary Figure 2. The responses were similar to those observed during PC activation.

### Effect of Autonomic Blockade on HR and BP Responses to IR Stimulation of the PC

**Figure 9:**
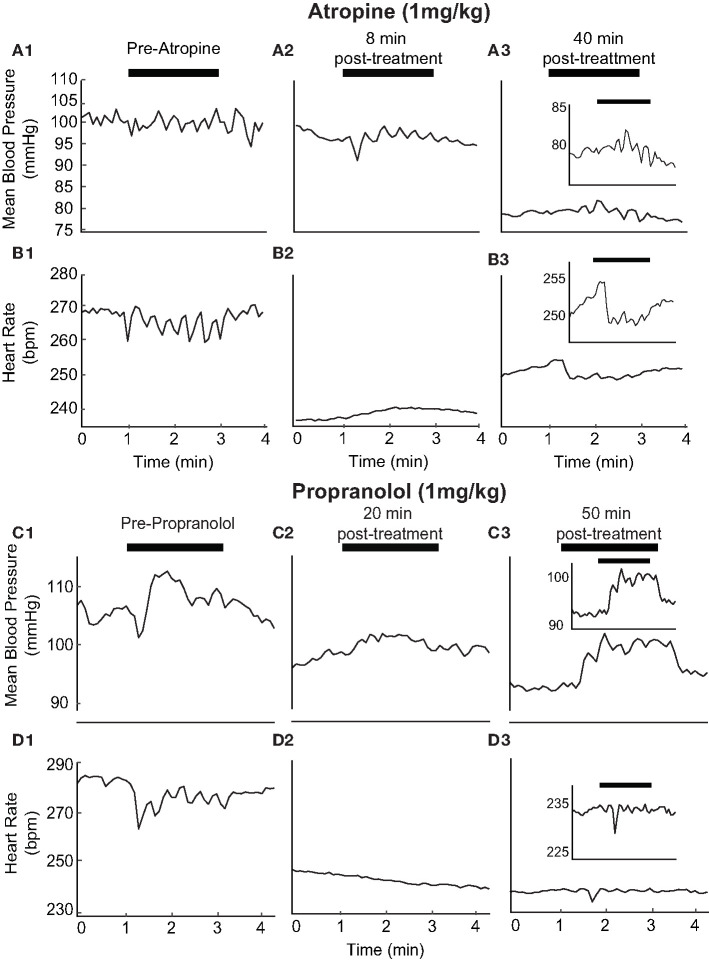
Autonomic blockers affect changes in BP and HR with activation of PC: The pharmacological blockers Atropine (1 mg/kg) (**A1–3** and **B1–3**) and Propranolol (1 mg/kg) (**C1–3** and **D1–3**) were used to block autonomic activity prior to IR stimulation. We observed BP and HR in response to IR stimulation of the PC prior to the injection of either drug consistent with previous results **(A1, B1, C1, D1)**. Treatment with either blocker led to alterations in IR evoked responses (Atropine: **A2, A3, Be, B3**, and Proporanalol: **C2, C3, D2, D3**). A more detailed view of recovering BP and HR responses can be seen for atropine and propranolol (insets). HR baselines reduced from 270 to 235 bpm with atropine and 285 to 245 bpm with propranolol. HR responses to stimulation are suppressed following administration of either drug and begin to return to baseline levels 40–50 minutes following injection.

The pharmacological blockade of vagal and sympathetic activities was achieved in naïve, additional rats by atropine (1 mg/kg) and propranolol (1 mg/kg) administration, respectively. The effects of these two drugs on BP and HR responses to unilateral pulsed IR stimulation of the PC were analyzed in separate experiments. The response after β-adrenergic receptor blockade with propranolol (1 mg/kg, iv) was used to estimate the sympathetic tone; the response after muscarinic cholinergic receptor blockade with methyl atropine (1 mg/kg, iv) was used to estimate the vagal tone (46). The initial changes induced by IR stimulation prior to the administration of atropine experiments was 2.54 ± 1.92 mmHg and 10.11 ± 6.52 bpm in the three rats tested (Figures 9A1,B1). Similarly, prior to the administration of propranolol, IR stimulation resulted in decreases of 3.46 ± 2.07 mmHg 15.40 ± 6.99 bpm (Figures 9C1,D1). Consistent with the effects of these drugs, we found a decrease in baseline HR by 21.83 ± 19.16 bpm following atropine administration and 88.99 bpm with propranolol (examples in Figures 9B2,D2). Recovery of HR was observed at ~50 min post-administration of atropine in most rats (Figure 9B3). IR stimulation of PC did not evoke a change in HR with administration of either drug in all three rats (Figures 9B2,D2). The IR evoked responses recovered 40–50 min after administration of both drugs (Figures 9B3,D3 and insets, for the example shown the HR recovered to 5.44 bpm post-atropine and 5.75 bpm post-proporanalol). In contrast, IR stimulation continued to evoke sinusoidal changes in BP after administration of atropine (Figures 9A2,A3, for the example shown the response was 5.49 mmHg), although these responses were eliminated following administration of propranolol (Figure 9C2). Responses from all three rats were characteristically similar.

## Discussion

In the present study, we used selective activation of either the PC or AC using pulsed IR to test a hypothesis that stimulation of a single vertical semicircular canal is sufficient to evoke a cardiovascular response. A subject-specific threshold level of radiant energy was necessary to elicit a cardiovascular response, above which there was a rapid decrease in HR and BP. Both measures then oscillated with the sinusoidal pulsed IR stimulation around the decreased plateau, returning to pre-stimulation baseline at the end of stimulation. The magnitude of the initial decrements in HR and BP did not correlate with the energy of the suprathreshold stimulus. This response pattern was consistent across multiple trials within an experimental session, replicable across subjects, and in most animals showed no evidence of habituation or an additive effect. Frequency modulated electrical current delivered to the PC caused decrements in HR and BP that resembled those evoked by IR stimulation of the PC and AC. Frequency domain HRV assessment revealed that, in most subjects, IR stimulation increased the low frequency component and decreased the high frequency component, resulting in an increase in the LF/HF ratio. Lastly, propranolol administration caused a substantial drop in baseline HR, and IR of the PC under these conditions did not evoke a change in HR or BP. In contrast, systemic atropine caused a small drop in baseline HR. IR of the PC during atropine treatment did not evoke a change in HR although stimulus-dependent sinusoidal modulations of BP were observed.

The brain requires stable cerebral perfusion in order to function normally and avoid syncope. One of the key physiological mechanisms mediating cardiovascular homeostasis is the baroreflex, a closed-loop negative feedback pathway deriving input from baroreceptors in the carotid body and aortic arch. When blood pressure increases, the associated distention of the arterial blood vessel walls is detected by these receptors, which convey excitatory signals via glossopharyngeal and vagal afferents to the caudal solitary nucleus (SolN) (47). SolN neurons relay the baroreflex-related input to GABAergic cells in the caudal ventrolateral medulla (CVLM), which project to pre-sympathetic cells of the adjacent rostral ventrolateral medulla (RVLM) (10, 48, 49). This inhibition reduces the level of activity in a series of barosensitive cell groups from RVLM, to preganglionic sympathetic neurons in the spinal cord, and ultimately the sympathetic trunk. Through this pathway, an increase in mean arterial pressure has been shown to result in a reduction in sympathetic nerve activity, relaxation of the vascular smooth muscle, and a reduction in arterial blood pressure due to baroreceptor unloading [for review, see Guyenet (50)].

Most of the vasculature receives innervation exclusively from sympathetic autonomic fibers, in a noradrenergic projection mediated by alpha adrenergic receptors. In contrast, HR is modulated by both sympathetic and parasympathetic fibers. The sympathetic fibers are noradrenergic, and their activation increases both contractility and relaxation rate of the heart muscles. The parasympathetic innervation originates from the dorsal motor vagal nucleus and nucleus ambiguus, and the cardiac ganglion cells receiving their input are cholinergic and activate muscarinic cholinergic receptors. In addition to its key role in mediating sympathetic outflow, SolN neurons can influence HR through projections to preganglionic parasympathetic cells in the nucleus ambiguus. Normally, the parasympathetic fibers restore cardiac homeostasis following sympathetic perturbation.

A large body of evidence from clinical observations and experimental studies in humans and animal models supports the notion that the vestibular system participates in the control of HR and BP during movement and postural adjustments (51). The redistribution of body fluids that occurs upon standing or rearing requires a rapid and proactive cardiovascular response in order to prevent blood from pooling in the legs and a subsequent decrease in cerebral perfusion (52). Since changes in head position and head motion activate the otolith organs and semicircular canals, the vestibular system is optimally situated to provide the requisite input to central cardiovascular circuits. This connection between the vestibular and autonomic systems allows for rapid modulation of HR and BP with changes in head position or body posture via vestibulo-autonomic pathways. Experimental studies in humans using various vestibular stimulation approaches have shown that activation of the vestibular labyrinth alters BP, blood flow, respiration and/or sympathetic nerve activity [for reviews, see Yates et al. (4); Hammam and Macefield (53); Yates and Miller (5)]. As highlighted in these reviews, considerable data exists showing that the vestibular inputs have complex effects on blood flow rates and pressure regulation to the head and hindlimbs and response patterning (54–56). Sinusoidally-modulated galvanic vestibular stimulation (sGVS) has been shown to evoke patterned sympathetic nerve activity in humans, specifically resulting in vasoconstriction of blood vessels in the legs (18, 20). This patterning of nerve activity resembles the sympathetic responses normally observed in humans during tilt and standing, and is thought to reflect an interaction between the baroreflex and the vestibular system (21). Moreover, head-up and head-down tilt delivered through off-vertical-axis rotation elicit up-and-down modulation of muscle sympathetic nerve activity that is in-phase with the oscillating stimulus (14). Since continuous off-vertical-axis rotation is thought to adapt the semicircular canals, this sympathetic modulation has been attributed to otolith end organ activation. There is little evidence until now from animal or human studies that stimulation of the vertical semicircular canals changes sympathetic nerve activity. However, studies on whole body oscillations in the yaw plane, which stimulate the horizontal semicircular canals, show a decrease in HR in humans (57). Warm and cool-water irrigation activate the horizontal canals and elicit caloric nystagmus, though its effects on sympathetic nerve activity and cardiovascular parameters in human subjects appear to be inconclusive (11, 58), for review, see Yates et al. (4).

There is a substantial literature demonstrating that sympathetic nerve activity changes during natural and electrical stimulation of the vestibular nerve in animals [for review, see Yates et al. (4)]. Following the initial seminal reports (24, 59), these studies generally indicate that head-up tilt and linear acceleration increase sympathetic nerve activity and raise BP (25, 29, 60), and that these increases are attenuated by bilateral peripheral vestibular damage (61). While the head-up tilts activate afferents from PC bilaterally, it is possible that our results when stimulating unilateral vertical canals reflect altered activity of vestibular nuclei neurons receiving convergent input from otolith and canals. A previous study has also shown that head-down tilt induced a rapid reduction in HR in normal subjects (62). Nose-down rotation causes a transient decrease in BP, which has also been attributed to vestibular input to autonomic pathways (28). Similar response was observed in patients with unilateral benign paroxysmal positional vertigo where the PC biomechanics is affected but only if the patients are tilted in the plane of the unaffected, contralateral PC (62). Future studies need to employ bilateral vertical canal stimulation to further characterize the observed responses. Furthermore, sGVS in rats can evoke drops in both BP and HR (29, 42, 63, 64). Most studies attribute the effects of sGVS, including those on autonomic activity, to otolith activation because of the low frequency characteristics of the stimuli and the perceptual, ocular, and postural responses that are elicited (18, 65). However, it has been suggested that the vertical canals may also be activated by the stimulus (4, 66, 67), raising the possibility that the vertical canals contribute to vestibulo-autonomic activity. In fact, both the otolith organs and semicircular canals can respond to tilt (17) and the vestibulo-ocular and -spinal reflexes can be evoked by combined otolith and canal input (60). We observed initial drops in HR with IR stimulation that were correlated with drops in BP and continued to modulate in-phase with the low frequency sinusoidal IR stimulation. These responses were replicable and stable over multiple trials, delivered over several hours. The magnitude of responses did not vary significantly over this prolonged experimental period. We did observe that the threshold IR radiant energy or strength of stimulus required to evoke a change in BP or HR varied (between 94 and 512 μJ/pulse) across the animals and was likely a result of small differences in orientation of the optical fiber between experiments. Overall, these results demonstrate that activation of a single vertical canal is sufficient to alter BP and HR. In future studies, it will be important to test contributions of the horizontal semicircular canals as well as stimulation of combined vertical canal and utricular macula.

The morphological basis for this vestibulo-autonomic activity has been demonstrated previously using sGVS to activate neurons in the caudal vestibular nuclei (42), and from there to brainstem regions involved in the central regulation of BP (9, 68–70) and HR (6, 71), as well as respiration (5, 72). These studies suggest that the vestibular system provides a means for rapid detection and autonomic activation in response to a postural change, requiring only ~100 ms to accomplish a change in BP following stimulation (10). This rapid change is followed by baroreflex activity, which re-establishes BP homeostasis 30–60 s after standing. Our results show that vertical semicircular canal activation induces a drop in both the HR and BP. This vaso-vagal-type response has also been reported following sGVS in rats (73), and can be attributed to the interaction between vestibulo-autonomic and baroreceptor reflexes. Supporting this interpretation, we observed that a measurable change in HR occurred prior to that of BP during IR stimulation. This observation highlights the non-linear nature of the relationship between HR and BP and suggests that the initial rapid cardiovascular response mediated by the vestibular system (14, 74, 75) is followed by baroreflex activity that has a substantially longer latency and as a result, may exacerbate or ameliorate the effects of the initial vestibulo-autonomic drive (76).

We used HRV analysis from the 17 rats (113 IR stimuli) receiving PC stimulation to further understand the effects of vertical canal stimulation on autonomic activity. HRV generally reflects the ability of the cardiovascular system to adapt to changing stimulus conditions and is used to assess fluctuations in autonomic innervation of the heart, as an alternative measure to average activity level. In general, healthy individuals have high HRV and reduced HRV is interpreted as an abnormal imbalance in the activity of the sympathetic and parasympathetic branches of the autonomic system (77). However, a decrease in HRV can reflect either withdrawal or excessively high activity in either of these branches. In the frequency domain, three components of HRV are distinguished: high, low and very low frequencies. The HF component can be viewed as predominantly an indicator of parasympathetic (vagal) activity. The LF component, however, reflects both the sympathetic and parasympathetic branches (78). The VLF component is typically a reflection of the stimulus. We found that IR stimulation caused an increase in the LF components with a corresponding decrease in HF components (Figure 7). This result is likely to reflect the interaction of short-latency vestibular effects on both sympathetic pathways through RVLM and on parasympathetic pathways through SolN, followed by cardiac and baroreflex efforts to re-establish homeostasis. In research using GVS, the HF component increased during stimulation and corresponded to increased mean arterial pressure in down-facing patients. The LF/HF ratio, a reflection of sympathetic nerve activity, increased with GVS in up-facing patients only (79). These results are consistent with studies using head-down neck rotation (80) and head-up off-vertical-axis rotation (14).

We further investigated the tonic sympathetic and vagal influences on the heart with selective pharmacological blockade of cardiac autonomic receptors. The ACh antagonist atropine was used to inhibit the effects of excessive vagal activation on the heart. The non-selective beta blocker propranolol works by blocking epinephrine and norepinephrine at β -adrenergic receptors. The response after β-adrenergic receptor blockade with propranolol was used to estimate the sympathetic tone; the response after muscarinic cholinergic receptor blockade with atropine was used to assess the vagal tone (46). Our results with IR following the pharmacological blocks suggest that the changes in HR evoked by IR are dependent on both sympathetic and parasympathetic activity but that BP responses are likely to be independent of parasympathetic activation.

### Conclusion

The present study demonstrates that activation of the vertical semicircular canals evokes significant autonomic activity in the anesthetized rats. The resulting decreases in MBP and HR confirm that these end organs provide a means for rapid detection and autonomic activation in response to a postural change. A direct comparison to the stimulation of unilateral otolith organs or augmentation of the responses seen here by otolith organs remain a goal for future studies.

## Data Availability Statement

The raw data supporting the conclusions of this article will be made available by the authors, without undue reservation.

## Ethics Statement

The animal study was reviewed and approved by Institutional Animal Care and Use Committee (IACUC) of the University of Miami.

## Author Contributions

DR, GM, GH, and SR conceptualized, designed the experiments, and prepared the manuscript. DR, GM, WJ, and SR carried out the experiments and collected the data. DR, GH, and SR analyzed the data and prepared all the figures. All authors approved the final manuscript.

## Conflict of Interest

The authors declare that the research was conducted in the absence of any commercial or financial relationships that could be construed as a potential conflict of interest.

## Supplementary Material

Supplementary Figure 1 Percent change in BP and HR. Percent changes in MBP (initial change in MPB/baseline MPB) and HR (initial change in HR/baseline HR) for each stimulation period for individual animals with PC stimulation (17 total with IR and 3 with electrical stimulation, outlined using dotted rectangle). *R*^2^-values are shown with each rat identification number.

Supplementary Figure 2 Magnitude of IR evoked changes in AC. The magnitude of changes in MBP (top) and HR (bottom) for 13 stimulation periods across four rats during the initial cycle of AC stimulation with IR are shown. **(A,B)** Show the responses to IR applied at varying radiant energies (377–512 μJ/pulse) when compared to the averaged baseline MBP (mmHg) (*R*^2^ = 0.078) and HR (bpm) (*R*^2^ = 0.083). **(C,D)** Show weak correlation between stimulation radiant energies (μJ/pulse) and the initial changes in MBP (mmHg) (*R*^2^ = 0.030) and HR (bpm) (*R*^2^ = 0.081). **(E,F)** show the relationship between consecutive periods and the magnitude of change in **(B1)** MBP (mmHg) (*R*^2^ = 0.174) and **(B2)** HR (bpm) (*R*^2^ = 0.118). Period numbers signify the number of events a single rat has undergone.
